# Effectiveness of multimodal exercises integrated with cognitive-behavioral therapy in working patients with chronic neck pain: protocol of a randomized controlled trial with 1-year follow-up

**DOI:** 10.1186/s13063-022-06340-7

**Published:** 2022-05-21

**Authors:** M. Monticone, S. Simone Vullo, L. I. Lecca, F. Meloni, I. Portoghese, M. Campagna

**Affiliations:** 1 Department of Medical Sciences and Public Health, University of Cagliari, Cagliari, Italy; 2 Neurorehabilitation Unit, Department of Neuroscience and Rehabilitation, G. Brotzu Hospital, Cagliari, Italy; 3 Department of Clinical Sciences and Translational Medicine, University of Rome Tor Vergata, Rome, Italy; 4 Doctoral Programme in Clinical Sciences, University of Florence, Florence, Italy

**Keywords:** Chronic neck pain, Rehabilitation, Task-oriented exercises, Cognitive-behavioral therapy, Randomized controlled trial

## Abstract

**Background:**

The etiology of neck pain is multifactorial and includes personal and work-related factors such as age, sex, wrong postures, and repeated strains. Studies based on bio-psychosocial model also link chronic neck pain to psychological factors. Over time, the use of multidisciplinary interventions for chronic neck pain has grown in order to improve disability, pain, and adaptive cognitions and behaviors towards patients’ problems. The objective is to evaluate the effectiveness of an individual-based multidisciplinary rehabilitation program that integrates cognitive-behavioral therapy focused on kinesiophobia with specific exercises in the treatment of patients with chronic neck pain, employed in different working activities.

**Methods:**

A randomized, parallel-group superiority-controlled trial will be conducted with 1-year follow-up. One hundred seventy patients engaged in several working activities (blue collar and white collar workers) will be randomly allocated to either the experimental (receiving a multidisciplinary rehabilitation program combining multimodal exercises with psychologist-lead cognitive-behavioral therapy sessions) or the control group (receiving general care physiotherapy). Both groups will follow individual-based programs once a week for 10 weeks. The main outcome measures will be the Neck Disability Index, the Tampa Scale for Kinesiophobia, the Pain Catastrophizing Scale, a pain numerical rating scale, the Short-Form Health Survey, and the Work Ability Index. Participants will be evaluated before, after training, and after 12 months.

**Discussion:**

Findings may provide empirical evidence on the effectiveness of an individual-based multidisciplinary rehabilitation program on inducing clinically significant and long-term improvements in the disability, pain, psychological factors, and quality of life of workers with chronic neck pain and that these would be maintained in the long term. Hence, this trial might contribute towards refining guidelines for good clinical practice and might be used as a basis for health authorities’ recommendations.

**Trial registration:**

ClinicalTrials.gov NCT04768790. Registered on 24 February 2021

## Administrative information

**Table:** 

Title	Effectiveness of Multimodal Exercises Integrated With Cognitive-behavioral Therapy in Subjects With Chronic Neck Pain: protocol of a Randomized Controlled Study With One Year Follow-up
Trial registration	ClinicalTrials.gov Identifier: NCT04768790; 02/24/2021
Protocol version	15 July 2021. Final Version
Funding	The author(s) won’t receive financial or non-financial support for the research, authorship, and/or publication of this article
Author details	Marco Monticone^1,2^, Salvatore Simone Vullo^1,3^, Luigi Isaia Lecca^1,4^, Federico Meloni^1^, Igor Portoghese^1^, Marcello Campagna^1^ Affiliations: 1 Department of Medical Sciences and Public Health, University of Cagliari, Cagliari, Italy 2 Neurorehabilitation Unit, Department of Neuroscience and Rehabilitation, G. Brotzu Hospital, Cagliari, Italy. 3 Department of Clinical Sciences and Translational Medicine, University of Rome Tor Vergata, Rome, Italy. 4 Doctoral Programme in Clinical Sciences, University of Florence, Florence, Italy
Name and contact information for the trial sponsor	Salvatore Simone Vullo s.simonevullo@gmail.com
Role of sponsor	N/A, the current study is investigator initiated and will not receive any financial support

## Introduction

### Background and rationale

Neck pain (NP) has been defined as discomfort or more intense forms of pain that are localized to the cervical region. This term generally refers to pain in the posterior or lateral regions of the neck. It has an episodic occurrence over a lifetime with variable degrees of recovery in between episodes. NP affects people of different age of both gender [1–3]. The estimated prevalence in a 12-month period is not uniform by working activity, but it varies from 20 to 58.5% [4, 5]. As previously reported by several scientific reports, several personal and occupational-related factors can influence the onset and the chronicity of NP, such as the presence of other musculoskeletal complaints, working in awkward/sustained postures, repetitive tasks, use of high-vibration vehicles, and poor self-rated health [7–8]. Low overall health has been found to have a role in the transition from acute to chronic pain and correlates with poor recovery from chronic pain [9, 10]. Moreover, psychosocial factors like fear of movement beliefs, catastrophising, and mood disorders seem to play a relevant role [11, 12], negatively interacting with physical, environmental; and occupational factors; contributing to reduce spinal mobility; and consequently having a detrimental effect on functional status, working activities, and the overall quality of life (QoL) [11, 12]. Also, neck pain and reduced mobility significantly impact work ability and occupational-related variables such as absenteeism and sick leave [13, 14].

In order to address improving strategies aimed to manage chronic low back pain and its consequences, multidisciplinary programs including exercises and cognitive-behavioral therapy (CBT) have been proposed, with significant results on reducing disability and pain, reverting maladaptive thoughts and behaviors, and enhancing the use of self-management skills [15–21]. Nevertheless, the effectiveness of combined physical exercise and CBT on determining long-lasting improvements of physical impairment, work limitations, pain, dysfunctional thoughts, and quality of life of subjects suffering from chronic NP is still questionable [22–26].

As a consequence, it is uncertain what kind of physical exercises for chronic NP can ensure better results between task-oriented and general exercises, the first addressed to functional recovery and independence in daily activities, and the second focused on increasing the range of motion and improving isometric muscle properties [27–29]. Moreover, it is still unclear how to enhance the effects of those exercises at medium and long term [27–29]. As reported in a systematic review [22], a wide range of CBT interventions for chronic NP, which includes relaxation, problem solving, reconditioning of maladaptive thinking patterns, management of fear-avoidance behaviors, and maladaptive coping strategies, has been used in the last years. Evidence supported effects in favor of CBT only on short-term pain relief when compared to no treatment, without any clinically meaningful effect; when comparing CBT to other interventions, or CBT in addition to another intervention vs. that intervention alone, no differences were found [22]. More clearly targeted interventions were therefore recommended to attain stronger and long-lasting treatment effects [30].

Hence, we planned a randomized parallel-group superiority-controlled trial aimed at evaluating the long-term effectiveness of a multidisciplinary program including individual-based task-oriented exercises (the aim of task-oriented exercises is the recovery of specific movements performed during job activities as well as early independence in ADL, based on the individual’s goals and personal needs; starting from the simplest form of exercise and proceeding with gradual increase in the complexity of tasks, this kind of exercises focuses on performing a functional task using verbal and visual feedback during practice [31]) and CBT focused on the management of fear-avoidance beliefs, catastrophizing, and maladaptive coping behaviors in comparison with individual-based general physiotherapy alone in the treatment of chronic NP. The primary hypothesis was that a 10-week multidisciplinary individual rehabilitation program including task-oriented exercises and CBT can result in clinically significant improvements in terms of reducing disability; the secondary hypothesis was that a 10-week multidisciplinary individual rehabilitation program can improve pain, psychological factors, QoL, and work ability in patients engaged in several working activities with chronic NP when compared with the individual-based general physiotherapy. Finally, we hypothesized that those improvements will be maintained at least 1 year.

## Methods: participants, interventions, and outcomes

### Trial design and study setting

A single-center, analyst-blind, two-arm, parallel-group, superiority, RCT including 170 patients engaged in several working activities (blue collar and white collar workers) with non-specific chronic NP will be conducted with 1-year follow-up in a secondary care rehabilitation hospital.

### Eligibility criteria

Inclusion criteria are as follows:

- Diagnosis of chronic non-specific (i.e., without a diagnosable cause) neck pain [32]
- Documented history of pain lasting more than 3 months
- Good knowledge of the Italian language
- Age over 18

Exclusion criteria are as follows:

- Acute (up to 4 weeks) and subacute (up to 12 weeks) neck pain
- Cognitive impairment (i.e., Mini Mental State Examination <24)
- Presence of specific causes of neck pain (history of spinal surgery, spinal deformity, herniated disc, infection, fracture, myelopathy or malignancy, whiplash injury, systemic or neuromuscular disease) evaluated by medical history and diagnostics for images
- Having previously undergone cognitive-behavioral therapy

### Participants

Between September 2020 and December 2021, patients referred to a secondary care rehabilitation hospital (Neurorehabilitation Unit, G. Brotzu Hospital, Cagliari, Italy) following an evaluation by two physiatrists coordinated by the principal investigator will be recruited at the point of care in respect of the inclusion criteria, receiving materials included in a booklet about the study and after declaring their consent to participate in the study, and they will be awarded by the physiatrists, through permuted-block randomization procedure with a 1:1 rate (an identification number will be assigned to each participant and listed in the dataset; treatment allocation will be automatically generated and concealed by the software Matlab), to one of the two treatment programs. Until interventions are assigned, the random sequence will be converted by the physiatrist in the list of participants assigned to each arm and provided to the physiotherapists and psychologist who will perform the treatment. In order to avoid a selection bias, physiatrists will strictly follow the inclusion and exclusion criteria and they will do only the first evaluation of the patients. The principal investigator remains blind to the allocation. Personal and occupational variables will be collected for enrolled patients.

A list of treatment codes and an automatic assignment system to conceal the allocation will be generated and stored through MATLAB. The principal investigator and whoever will carry out the analysis of the data will be blinded to treatment allocation. The physiotherapists, the psychologist, and the patients will not be blinded.

### Intervention description

The interventions will be performed by two physiotherapists with equal experience (10 to 15 years’ experience in spinal disorders; before the study begun, they received an adequate training, performed by the physiatrists, on the treatment under the protocol), each one responsible for one of the two branches (multidisciplinary and general exercise group). In addition, for the multidisciplinary group, a psychologist (10 to 15 years’ experience in chronic pain management who did not receive any specific training as part of the study) will perform individual CBT sessions. The following evaluations will be performed for each patient of the two groups, by the assigned physiotherapist: postural observation, cervical range of motion examination, and muscle strength. The exercises will be planned based on the previous evaluation. Following the Italian National Health System protocols on physiotherapy sessions, 10 physical training sessions of 60 min once a week for 10 weeks will be programmed. Additionally, patients will repeat the exercises at home. In order to improve daily activities, during the first session each patient will be provided with ergonomic advice through a booklet. At the end of the treatment, patients will be asked to continue with the exercises at home and they will be also asked to rate the effectiveness of the treatment using the 5-point Likert Global Perceived Effect scale (1=helped a lot, 2=helped, 3=helped only a little, 4=did not help, 5=made things worse) [33]. Based on a manual that includes the list of all the exercises to be delivered, at the end of each session a check of the program performed by the physiotherapist will be conducted, in order to verify that all the planned exercises will be actually performed. During the program intervention, no other treatment will be performed (e.g., physical modalities, nerve blocks, drug therapies), while family doctors will be asked to avoid referring patients to other visits and / or other treatments. Using a specific form, patients will be asked to report any serious symptoms they have during the trial. Participants will be inquired during each session and at the follow-up about their satisfaction with the home-exercises; any reasons for skipping home exercises, seeking additional care, or taking medications to control pain will be discussed. In case of a condition that precludes further participation in the trial or the loss of eligibility (e.g., serious adverse events or other complications; subjects with poor compliance that are unable to complete the entire set of exercises; subjects unable to continue with the clinical trial and request the investigator to withdraw), the participant will be discontinued from trial participation with full disclosure of the reasons and the principal investigation could be informed.

#### Multidisciplinary program interventions

Multimodal exercises will be introduced to improve, through gradual exposure, cervical mobility, postural control, and strengthening of the cervical muscles. Patients will learn stabilizing techniques for neck deep muscles, progressively increasing the speed and complexity of the movements. Task-oriented exercises will then be introduced while maintaining the activation of the deep spinal muscle. Under the supervision of the psychologist, the subjects will also be involved in cognitive-behavioral therapy aimed at modifying fear of movement, catastrophizing, and maladaptive illness behavior on the basis of the fear-avoidance beliefs, the administered questionnaires, and the presentation of images showing neck-stressing activities. Each patient assigned to the multidisciplinary group will meet with the psychologist once a week for a 60-min individual session of CBT. The multidisciplinary program will last for a total of 20 h.

#### General program interventions

General physiotherapy will include exercises for spinal muscle strengthening, regional stretching, and mobilization. The general exercise program will last for 10 h.

### Outcomes

#### Primary outcome measure

##### Change from Baseline Neck Disability Index at 10 weeks and 12 months (mean)

Neck Disability Index [34]: it is a self-administered 10-item questionnaire concerning NP disability and rates the intensity of pain and its disabling effects on typical daily activities. The score, expressed as a percentage, ranges from 0 (no disability) to 100 (maximum disability). Investigators will use the Italian version which proved to be reliable and valid [35].

#### Secondary outcome measure

##### Change from Baseline Tampa Scale of Kinesiophobia at 10 weeks and 12 months (mean)

Tampa Scale of Kinesiophobia [36]: it is a self-report questionnaire that assesses pain beliefs and pain-related fear of movement/reinjury in subjects with musculoskeletal complaints. The 13-item Italian version ranges from 13 (best health status) to 52 (worst health status).

##### Change from Baseline Pain Catastrophizing Scale at 10 weeks and 12 months (mean)

Pain Catastrophizing Scale [37]: it assesses catastrophizing in subjects with musculoskeletal complaints and consists of a 13-item self-report questionnaire. The total score ranges from 0 to 52 with 52 indicating the worst health status.

##### Change from Baseline Numerical Rating Scale at 10 weeks and 12 months (mean)

Numerical Rating Scale [38]: it assesses pain intensity using an 11-point numerical rating scale ranging from 0 (no pain) to10 (the worst imaginable pain).

##### Change from Baseline Short-Form Health Survey Questionnaire at 10 weeks and 12 months (mean)

Short-Form Health Survey Questionnaire: quality of life is assessed using the Italian version of the self-report Short-Form Health Survey [39, 40] with its eight domain scores ranging from 0 (the worst perceived quality of life) to 100 (the best perceived quality of life).

##### Change from Baseline Work Ability Index at 10 weeks and 12 months (mean)

Work Ability Index [41]: it is a tool used in occupational health care and research to assess work ability of workers exploring different dimensions; Work Ability Index is scored by summing the points received for each item: the best possible rating on the index is 49 points and the worst is 7 points.

All of the outcome measures will be administered before rehabilitation, at the end of the rehabilitation programs (10 weeks) and at follow-up (12 months post-rehabilitation, considered as a conventional long-term follow-up commonly used in rehabilitative studies).

### Participant timeline

**Fig. 1:**
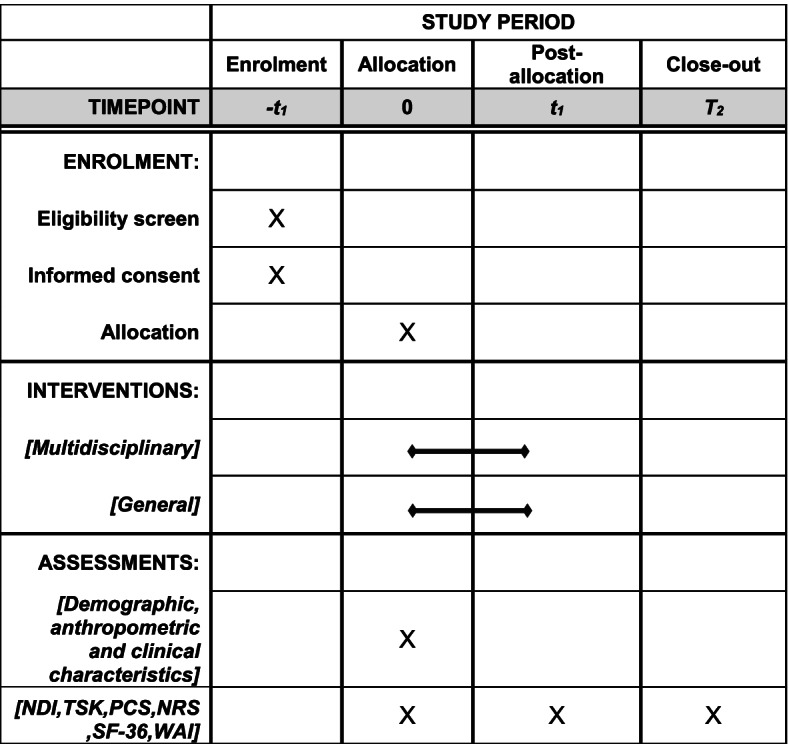
Schedule of enrolment, interventions, and assessments. NDI, Neck Disability Index; TSK, Tampa Scale of Kinesiophobia; PCS, Pain Catastrophizing Scale; NRS, Numerical Rating Scale; SF-36, Short-Form Health Survey Questionnaire; WAI, Work Ability Index

The participant timeline is shown in Fig. 1.

## Data collection and management

The principal investigator will obtain and assess the data and the biostatistician will make the analyses.

Pursuant to the European Regulation on the Protection of Personal Data (GDPR 2016/679), articles 5 (Principles applicable to the processing of personal data), 6 (lawfulness of processing), and 7 (conditions for consent), the data collected will be used exclusively for scientific research purposes. All information collected will be stored securely and prevented from being viewed by outsiders. Any information that could identify the participants will be removed to ensure their anonymity. An identification number will be assigned to each participant and listed in the dataset. The material will be kept by the principal investigator.

## Sample size and statistical analysis

The sample size will be based on the Italian Neck Disability Index, for which it will be estimated that a between-group difference of 7 points should be considered as clinically important [34]. The sample size is estimated at 154 patients, in order to ensure 5% type I error and 20% type II error and considering a standard deviation ± 15.4. However, considering a dropout of 10%, 170 patients will be recruited. Mann-Whitney *U* test will be used to analyze the differences between the two groups relating to measures with non-normal distribution. Linear mixed-effects models for repeated measures (*p*<0.05) will be used to compare mean changes in primary and each secondary outcome between intervention and control group of patients over time on an intention to treat basis [42, 43]. The null hypothesis will be rejected for *p* values> 0.05 (two-tailed test).

All statistics will be done using SPSS 21.0 (IBM SPSS Statistics for Windows, Version 21.0, Chicago, IL, USA) software. Results for normally distributed continuous variables will be expressed as the mean value ± standard deviation, and continuous variables with nonnormal distribution will be presented as median values and interquartile range. Demographic and baseline characteristics will be summarized with the use of descriptive statistics. Categorical variables will be reported as absolute numbers and percentages.

### Discontinuation criteria and treatment

- Subjects who experience serious adverse events or other complications, and who, in the judgment of the investigator, should be discontinued from the trial and given appropriate treatment.
- Subjects who have poor compliance and are unable to complete the entire set of exercises, which have an impact on the study results.
- Subjects who, for whatever reason, are unwilling or unable to continue with the clinical trial and request the investigator to withdraw from the trial and discontinue the trial.

For discontinued cases, noncompliance, and/or loss to follow-up, missing data analysis will be performed to identify the patterns in missing variables. Specifically, we will assess whether missing values are likely to be missing at random (MAR), missing completely at random (MCAR), or missing not at random (MNAR). In the event of MCAR or MAR data, we will use multiple imputation techniques and present the outcome of analyses with and without the imputed data. In the event of MNAR data, we will not replace missing values and we will conduct case analysis when available. In linear mixed-effects models for repeated measures, unbiased estimation of the treatment effect will be adjusted for time-varying confounders.

*T*-test of independent samples for age, pain duration, body mass index, and outcome measures at baseline and Pearson chi-squared test for categorical variables (gender, occupation and type of exercises) were used to compare participants’ characteristics at baseline between groups. Linear mixed model analyses for repeated measures (significance level of 5%) were conducted for each of the measures, with group and time used as fixed effects, the outcome measure as dependent variable, and the participants as random effect. The time by group interaction term was also evaluated. Between-group differences at the end of the program and at follow-up were evaluated for the primary outcome and the other variables.

At the time of submission of the study, 59 (35% of the sample size) patients have already been recruited and related data collected.

## Auditing

An auditing will be performed at the end of the treatment and at the end of the follow-up, and they will be chaired by the physiatrists. The principal investigator will not participate at the auditing, ensuring the independence of the analysis.

## Dissemination plans

All scientific information derived from this trial will be published, possibly in national and international scientific journals with a peer-reviewing system, and the intellectual property of the data will be of the Department of Medical Sciences and Public Health of the University of Cagliari and of the investigators who will be free to publish the data, present them to Congresses, and disseminate them in scientific and public venues deemed most useful for the dissemination of the acquired knowledge.

## Discussion

Chronic NP is a common musculoskeletal problem which causes ADL and work difficulties, disability, and economic and social costs for both patients and society [3, 44–47].

Although it is already known that multidisciplinary programs that include exercises and cognitive-behavioral therapy (CBT) can lead to clinically important and long-lasting changes in physical impairment, work limitations, pain, dysfunctional thoughts, and quality of life in subjects with chronic low back pain [15–21], it is still questioned if similar programs that comprise exercises and CBT may have the same results on chronic NP [23–27].

Some limitations affect the present trial. First, data monitoring committee (DMC) is not provided. Given the short-term duration of the follow-up (12 months) involving low risks for patients under treatment and without critical safety concerns, mainly evaluating relief of symptoms as outcome, the DMC can be omitted without any risk for patients and the results of the trial.

The study is expected to provide new data on the effectiveness of a multidisciplinary rehabilitation program on inducing clinically significant and long-term improvements in the disability, pain, psychological factors, quality of life, and work ability of patients with chronic NP engaged in different working activities and that these would be maintained in the long term, compared to general physiotherapy.

Hence, this trial might contribute towards refining guidelines for good clinical practice and might be used as a basis for health authorities’ recommendations.

## Trial status

Protocol final version—July 15 2021: recruitment started on September 2020 and approximately will be completed on December 2021.

## Data Availability

The datasets of this study will be not publicly available due to individual privacy rules; only the principal investigator can access the blinded dataset, as well as biostatistics in order to perform data analysis. Anonymized dataset and statistical code will be made available under motivate request at the corresponding author. Only the principal investigator can share the requested data, after the evaluation of the motivation provided by the claimant.

## Abbreviations

ADL: Activity of daily living
CBT: Cognitive-behavioral therapy
NP: Neck pain
